# Mechanisms of multiple neurotransmitters in the effects of Lycopene on brain injury induced by Hyperlipidemia

**DOI:** 10.1186/s12944-018-0660-5

**Published:** 2018-02-07

**Authors:** Weichun Yang, Ziyi Shen, Sixian Wen, Wei Wang, Minyu Hu

**Affiliations:** Department of Nutrition and Food Hygiene, Xiangya School of Public Health, Central South University, 110 Xiangya Road, Changsha, 410078 China

**Keywords:** Lycopene, Hyperlipidemia, Brain injury, Neurotransmitters, Mechanism

## Abstract

**Background:**

Lycopene is a kind of carotenoid, with a strong capacity of antioxidation and regulating the bloodlipid. There has been some evidence that lycopene has protective effects on the central nervous system, but few studies have rigorously explored the role of neurotransmitters in it. Therefore, the present study was designed to investigate the effects of several neurotransmitters as lycopene exerts anti-injury effects induced by hyperlipidemia.

**Methods:**

Eighty adult SD rats, half male and half female, were randomly divided into eight groups on the basis of serum total cholesterol (TC) levels and body weight. There was a control group containing rats fed a standard laboratory rodent chow diet (CD); a hypercholesterolemic diet (rat chow supplemented with 4% cholesterol, 1% cholic acid and 0.5% thiouracil – this is also called a CCT diet) group; a positive group (CCT + F) fed CCT, supplemented with 10 mg·kg·bw^− 1^·d^− 1^ fluvastatin sodium by gastric perfusion; and lycopene groups at five dose levels (CCT + LYCO) fed with CCT and supplied lycopene at doses of 5, 25, 45, 65, and 85 mg·kg·bw^− 1^·d^− 1^. The levels of TC, triglyceride (TG), low-density lipoprotein cholesterol (LDL-C), high density lipoprotein cholesterol (HDL-C), interleukin-1 (IL-1), tumor necrosis factor alpha (TNF-α), oxidized low density lipoprotein (ox-LDL), low-density lipoprotein receptor (LDLR), nerve growth factor (NGF), glutamic acid (Glu), Gamma aminobutyric acid (GABA), dopamine (DA), 5-hydroxytryptamine (5-HT), N-methyl-D-aspartate (NMDA1R), GABA_A_, 5-HT_1_, D_1_, and apoptosis-related proteins Caspase3, bax, and bcl-2 were measured after the experiment. Nissl staining was adopted to observe the morphological changes in neurons.

**Results:**

At the end of the experiment, the levels of TC, TG, LDL-C, IL-1, TNF-α, and ox-LDL in the serum and brain as well as the content of Glu, DA, NMDA, and D_1_ in the brain of rats in the CCT group were higher than those in the control group (*P*<0.05); the levels of LDLR, NGF, GABA, 5-HT, GABA_A_, 5-HT_1_, and neuron quantities in the hippocampal CA1 and CA3 areas were lower than those in the control group (*P*<0.05). Compared to the CCT group, levels of TC, TG, LDL-C, IL-1, TNF-α, and ox-LDL in the serum and brain, as well as the content of Glu, DA and the expression of pro-apoptotic Caspase3 in the brain decreased in the rats with lycopene (25 mg to 85 mg) added to the diet (*P*<0.05); the levels of LDLR, NGF, GABA, 5-HT, GABA_A_, and 5-HT_1_ as well as the expression of anti-apoptotic bcl-2 and the neuron quantity in hippocampal CA1 and CA3 areas increased (*P*<0.05); further, the hippocampal cells were closely arranged. Lycopene dose was negatively correlated with the levels of TC, TG, and LDL-C in the serum and brain as well as levels of IL-1, TNF-α, ox-LDL, Glu/GABA, NMDA1R, and Caspase3 (*P*<0.05); it was positively correlated with the levels of LDLR, NGF, 5-HT, 5-HT_1_, GABA_A_, bcl-2, and the neuron quantity in hippocampal CA1 and CA3 areas (*P*<0.05).

**Conclusions:**

Lycopene exerts anti-injury effects in the brain as-induced by hyperlipidemia. It can inhibit the elevation of serum TC, TG, and LDL-C in rats with hyperlipidemia while indirectly affecting the levels of TC, TG, and LDL-C in the brain, leading to a reduction in ox-LDL, IL-1, and TNF-α in the brain. This inhibits the release of Glu, which weakens nerve toxicity and downregulates pro-apoptotic Caspase3. Lycopene also plays an anti-injury role by promoting the release of the inhibitory neurotransmitter GABA and 5-HT, which enhances the protective effect, and by upregulating the anti-apoptotic bcl-2.

## Background

Hyperlipidemia is not only a major risk factor for atherosclerosis, coronary heart disease, and ischemic stroke [1, 2], but is also closely related to neurodegenerative diseases such as Alzheimer’s disease (AD) [3, 4], Parkinson’s disease [5], and anxiety [6, 7]. A Framingham prospective cohort study suggested that obesity is a risk factor for cognitive impairment; studies have also shown that a hyperlipidemia can lead to inflammatory damage to the central nervous system resulting in cognitive disorders [8]. The central neurons system is composed of neurons and glial cells and mainly relies on the neurotransmitter for information transmission. Any disorder of neurotransmitter metabolism can lead to nervous system disease [9, 10]. Hyperlipidemia can destroy the balance of neurotransmitters in the brain [11, 12], affect the metabolism of brain substances and energy, and thus damage overall cognitive function [13]. Fluvastatin, one of the generally accepted drugs for treatment of hyperlipidemia, bases its effects on antioxidation and slowing the production of cholesterol in the body, but with sizable costs and risk for side effects [14].

Lycopene is a kind of carotenoid containing a carbon skeleton of about 40 atoms with an unsaturated straight hydrocarbon chain comprised of 11 conjugated and 2 non-conjugated carbon-carbon (C=C) unsaturated bonds [15]. It can readily quench singlet oxygen, scavenge free radicals, enhance immunity, promote information transmission [16], and inhibit tumor proliferation [17, 18]. Epidemiological studies have shown that lycopene can reduce mortality in patients with AD [19], and that serum lycopene concentration is closely related to cognitive dysfunction [20]. Early studies indicate that lycopene can effectively lower levels of serum TC, TG and LDL-C of rabbits with artherosclerosis induced with high fat and inhibit atherogenesis with an effect equivalent to that of fluvastatin [21]. Hyperlipidemia can affect the lipid metabolism in the brain of rats [22], disrupt the structure of hippocampal neurons, and damage cerebral blood vessels and neurons [23]. Lycopene can protect hippocampal neurons in a variety of ways, and has shown protective effect on the neuron injury induced by hyperlipidemia in rats [24]. Nervous system diseases induced by hyperlipidemia are closely related to neurotransmitters. The aim of the present study was to investigate the mechanism of lycopene on brain neurotransmitters in exerting anti-injury effects with hyperlipidemia, and to provide experimental evidence for the prevention and treatment of neurodegenerative diseases.

## Methods

### Composition of diets

The standard diet given to the rats consisted of the following ingredients: 20% wheat, 20% rice, 10% corn, 24% soybean cake, 10% fish flour, 10% wheat bran, 1% salt, 3% bone meal, and 1% multivitamins; the diet was purchased ready-made from the Department of Zoology, Xiangya School of Medicine, Central South University. The a hypercholesterolemic diet consisted of 94.5% of the standard diet plus 4% cholesterol, 1% cholic acid, and 0.5% thiouracil – this is also called a CCT diet [25], all purchased from Anhui Apocalypse Chemical Technology Co., Ltd. Lycopene was purchased from North China Pharmaceutical Co., Ltd. with purity of 93%.

### Experimental animals and design

Eighty healthy adult Sprague-Dawley rats with a body weight of 222.52 ± 10.39 g, half male and half female, were purchased from the Department of Zoology, Xiangya School of Medicine, Central South University. All experimental procedures were conducted in accordance with the guidelines of the animal ethical committee for animal experimentation in China, and the experimental design was approved by the Xiangya School of Public Health IRB (XYGW-2015-03). After a one-week acclimatization period, the animals were distributed into eight experimental groups of ten rats each based on their measured levels of total cholesterol (TC). Group I (CCT group) rats were fed a CCT to induce hyperlipidemia for 4 weeks. Group II (control group) rats were fed a standard laboratory rodent chow as a control diet (CD) for 4 weeks. Group III (CCT + F) was a positive drug treatment control group, rats were fed the CCT plus fluvastatin sodium at dose of 10 mg·kg·bw^− 1^·d^− 1^ for 4 weeks. Group IV, V, VI, VII, and VIII (CCT + LYCO) animals were fed the CCT and supplied with various doses of lycopene (5, 25, 45, 65, and 85 mg·kg·bw^− 1^·d^− 1^, respectively). The rats in the CCT and control group were gastrogavaged with 1% carboxymethylcellulose sodium (CMC-Na) solvent. Fluvastatin sodium and lycopene powder with 1% CMC-Na as the solvent were administrated to animals in other groups at the designated doses (with gastric administration volume of 1 ml·d^− 1^ for rats in all groups) for 4 weeks. The rats were raised in separate cages with daylight illumination and given water ad libitum. The temperature in the animal room was controlled at 24 ± 2 °C and the humidity at 65–70%. The food intake was recorded everyday and, based on the consumption of normal rats in a pilot experiment, restricted to 20 ± 2 g daily in order to prevent excessive weight gain.. The rats were weighed twice weekly and the gastric administration volumes of fluvastatin sodium and lycopene were adjusted according to the body weights.

### Detection methods and quality control

At the ends of week 0, week 1, week 3 and week 4 of the experiment, the animals were fasted for 12 h and their tails were cut to collect blood samples for serum TC, Triglyceride (TG), density lipoprotein cholesterol (LDL-C), and high density lipoprotein cholesterol (HDL-C) level assays. At the end of the experiment, the rats were weighed before the they were euthanized via an i.p. injection of sodium pentobarbital (45 mg·kg·bw^− 1^). Blood samples were obtained from the sacrificed rats from the abdominal aorta. Following a period of 30 min at room temperature, the samples were centrifuged at 3000 g for 10 min and the serum was separated, placed into Eppendorf tubes, and stored at − 20 °C until determination of the biochemical parameters. The brain tissue was excised and rinsed in ice-cold normal saline to remove blood and other extraneous substances, dried on a filter paper, and weighed. The brain was dissected bilaterally and one hemisphere was used for morphology analysis: The brain samples were homogenized in a basic solvent deemed appropriate according to the biochemical indicators (10 volumes of phosphate-buffered saline for biotin-avidin-based enzyme-linked immunosorbent assay (ELISA) or methanol for lipid detection), then centrifuged at 1000 g for an 15 min at 4 °C. The obtained supernatant was used for all subsequent analyses on the brain tissue. The other hemisphere was fixed in 4% paraformaldehyde solution, then a tissue piece about 1 cm thick was cut in the direction of the procerebrum and imbedded with paraffin. The coronal plane was cut to produce three sections 4 μm in thickness to take biochemical measurements from each rat.

The levels of TC and TG in the serum and brain were measured using enzymatic methods of cholesterol oxidase-peroxidase-4-aminoantipyrine (COD-PAP) and glycerol phosphate oxidase-peroxidase-4-aminoantipyrine (GPO-PAP), respectively [26]. LDL-C and HDL-C were detected by double reagent direct method [27]. The concentrations of interleukin-1 (IL-1), tumor necrosis factor alpha (TNF-α), oxidized low density lipoprotein (ox-LDL), low density lipoprotein receptor (LDLR), nerve growth factor (NGF), glutamic acid (Glu), gamma aminobutyric acid (GABA), 5-hydroxytryptamine (5-HT), and dopamine (DA) in the brain were all determined using ELISA. Western blot analysis was used to determine the levels of neurotransmitter receptors N-methyl-D-aspartate (NMDA1R), GABA_A_, 5-HT_1_, and D_1_, as well as apoptosis-related proteins Caspase-3, bax, and bcl-2 in the brain samples. Morphological changes in the hippocampal neurons were evaluated using Nissl staining.

The instruments were debugged and calibrated before use and a practice run with distilled water was performed before the experiment itself. Lycopene was dissolved in 1% CMC-Na and the gastric administration solution was freshly prepared before use. All testing was implemented in accordance with any and all manufacturer’s instructions, and the experimental data was double-recorded, input, and checked.

### Statistical analysis

All analyses were carried out in SPSS 18.0 software. Quantitative data is expressed here as mean ± standard deviation. Normality tests and homogeneity of variance tests were performed. Analysis of variance was applied to repeated measurement data to test the difference of blood lipid levels in different groups of rats at different times. Differences among groups were analyzed using ANOVA or Kruskal-Wallos H tests followed by post hoc Student-Newman-Keuls tests or Dunn-Bonferroni method. Pearson correlation or Spearman rank correlation analysis was used for correlation analysis and the R Programming Language software was used to draw the correlation coefficient matrix of serum lipid and brain parameters. Probability values of less than 5% (*P* < 0.05) were considered significant.

## Results

**Fig. 1:**
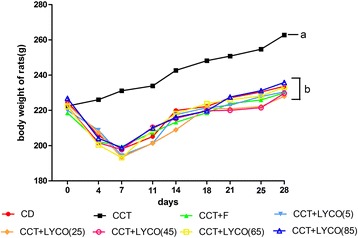
The body weight of rats in different groups at different time. *n* =10, values are expressed as the mean ± SD, and lines at the end of week 4 point without a common superscript letter differ significantly (*P* < 0.05). *F*_time_ = 125.341, *F*_groups_ = 8.262, *F*_time·groups_ = 2.209, *P* = 0.000

During the experiment, the rats performed well in terms of water drinking, diet consumption, and defecation; their body weight changes and activities were all normal. Analysis of variance of repeated measurement of body weight results of each group are shown in Fig. 1.

No significant difference was observed in body weight in the groups fed the CCT at any point in the study (*P* > 0.05). Analysis of variance of repeated measurements showed that there were significant differences in body weight in the animals in different groups at various stages of the experiment (*P* < 0.05).

### Serum lipid measurement

**Fig. 2:**
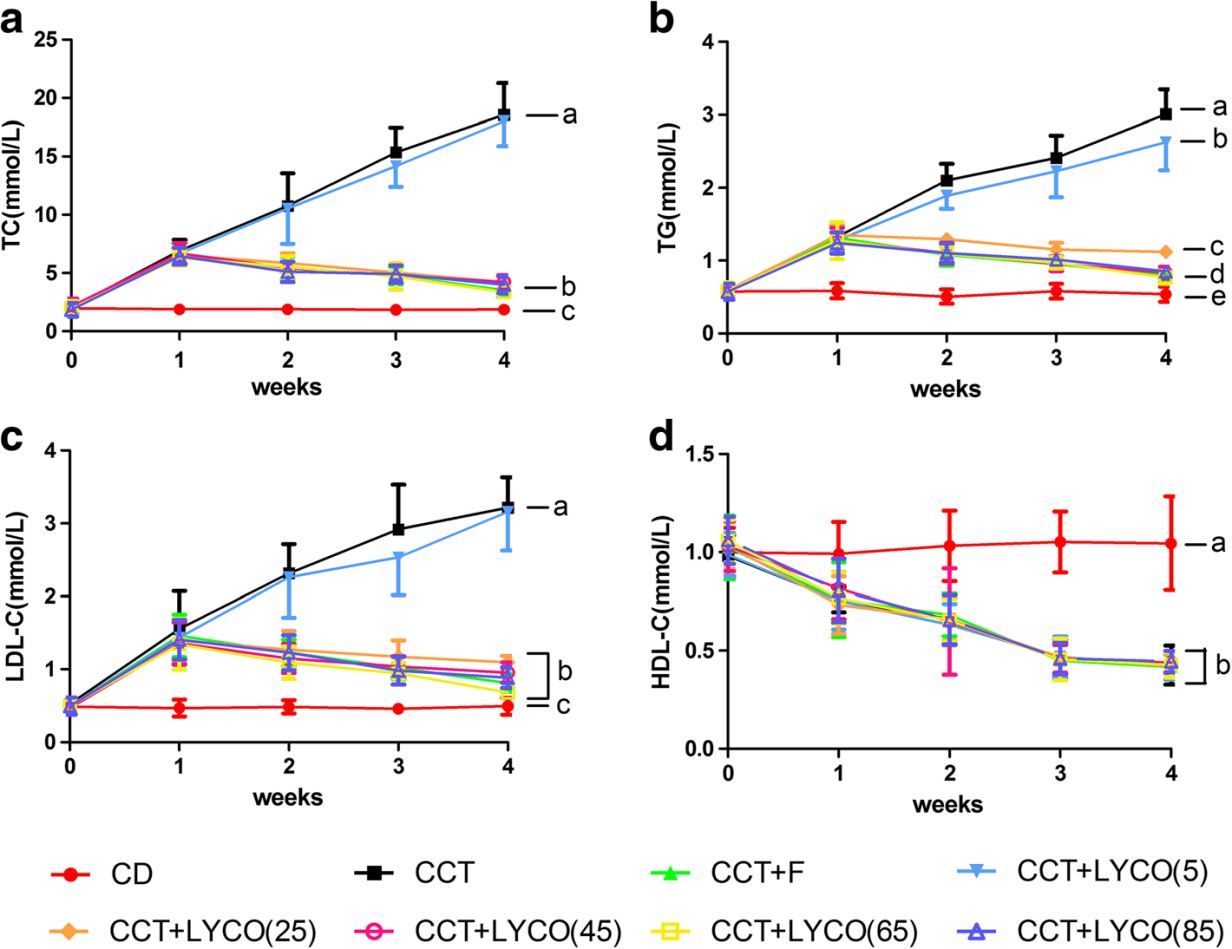
Serum lipid in different groups at different time. *n* =10, values are expressed as the mean ± SD, and lines at the end of week 4 point without a common superscript letter differ significantly (*P* < 0.05). Repeated measures analysis of variance and Kruskal-Wallis *H* test results at the end of the 4th week showed: **a** TC in serum: *F*_time_ = 498.078, *F*_groups_ = 130.034, *F*_time·groups_ = 119.999, *P* = 0.000; *H*_4weeks_ = 64.344, *P* = 0.000. **b** TG in serum: *F*_time_ = 396.967, *F*_groups_ = 191.878, *F*_time·groups_ = 84.739, *P* = 0.000; *H*_4weeks_ = 69.784, *P* = 0.000. **c** LDL-C in serum: *F*_time_ = 181.344, *F*_groups_ = 154.224, *F*_time·groups_ = 33.943, *P* = 0.000; *H*_4weeks_ = 70.338, *P* = 0.000. **d** HDL-C in serum: *F*_time_ = 252.260, *F*_groups_ = 31.848, *F*_time·groups_ = 6.617, *P* = 0.000; *H*_4weeks_ = 27.366, *P* = 0.000

Analysis of variance of repeated measurement of serum lipid results of each group at the end of week 0, week 1, week 3 and week 4 of the experiment are shown in Fig. 2.

As shown in Fig. 2, there were significant differences in rat serum TC, TG, LDL-C, and HDL-C (*P* < 0.05). At the end of the first week of the experiment, the serum TC, TG, and LDL-C of rats fed the CCT increased significantly, by 3.40, 2.29, and 3.00 fold (*P* < 0.05) while that of HDL-C was reduced 0.78 fold (*P* < 0.05). With prolonged feeding time, the levels of serum TC, TG, and LDL-C were significantly increased in CCT and CCT + LYCO (5 mg·kg^− 1^) groups (*P* < 0.05) but significantly decreased in CCT + LYCO (25, 45, 65, and 85 mg·kg^− 1^) groups (*P* < 0.05). At the end of the experiment, the levels of serum TC, TG, and LDL-C in CCT + LYCO (25, 45, 65 and 85 mg·kg^− 1^) and CCT + F groups and the levels of serum TG in the CCT + LYCO (5 mg·kg^− 1^) group were significantly reduced compared to the CCT group (*P* < 0.05). Compared to the control group, serum HDL-C decreased in other groups (*P* < 0.05) but there was no significant difference between them (*P* > 0.05).

### Brain lipid measurement

**Fig. 3:**
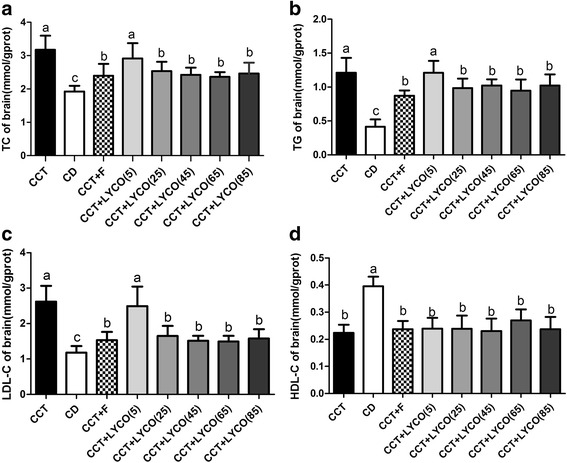
Brain lipid in different groups at the end of the experiment. *n* =10, values are expressed as the mean ± SD, and bars without a common superscript letter differ significantly (*P* < 0.05). Kruskal-Wallis *H* test or ANOVA results showed: **a** TC in brain: *H* = 43.211, *P* = 0.000; **b** TG in brain: *H* = 47.247, *P* = 0.000; **c** LDL-C in brain: *H* = 52.070, *P* = 0.000; **d** HDL-C in brain: *F* = 19.981, *P* = 0.000

At the end of the experiment, The brain lipid results are shown in Fig. 3.

Compared to the CD group, the levels of brain TC, TG and LDL-C increased significantly to 1.65, 2.93 and 2.22 times (*P* < 0.05), HDL-C decresed to 0.57 times (*P* < 0.05). CCT and CCT + LYCO (5 mg·kg^− 1^) groups did not show any significant difference on the levels of brain TC, TG and LDL-C (*P* > 0.05). Compared to the CCT group, e levels of brain TC, TG and LDL-C in CCT + LYCO (25, 45, 65 and 85 mg·kg^− 1^) and CCT + F groups were significantly reduced (*P* < 0.05). And brain HDL-C decreased in the groups fed a CCT regardless of fluvastatin sodium or lycopene administered (*P* < 0.05), but there was no significant difference between them (*P* > 0.05).

### The oxidative and inflammatory indices

**Fig. 4:**
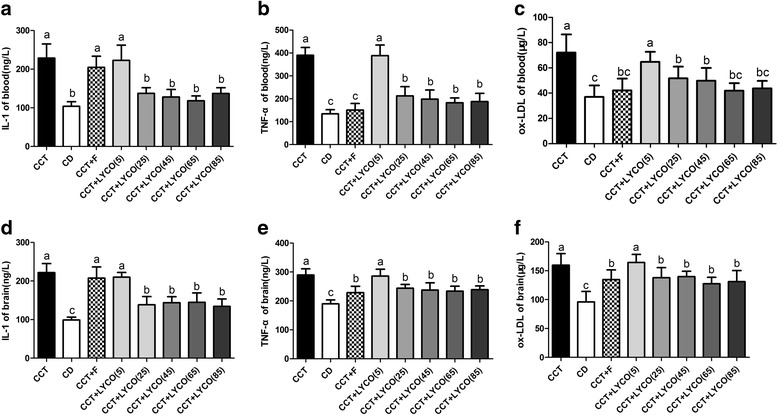
Serum and brain IL-1, TNF-α and ox-LDL in different groups at the end of the experiment. *n* =10, values are expressed as the mean ± SD, and bars without a common superscript letter differ significantly (*P* < 0.05). Kruskal-Wallis *H* test or ANOVA results showed: **a** IL-1 in blood: *H* = 63.526, *P* = 0.000; **b** TNF-α in blood: *H* = 59.785, *P* = 0.000; **c** ox-LDL in blood: *F* = 16.971, *P* = 0.000; **d** IL-1 in brain: *H* = 64.063,*P* = 0.000; **e** TNF-α in brain: *H* = 54.703, *P* = 0.000; **f** ox-LDL in brain: *F* = 16.829, *P* = 0.000

The levels of IL-1,TNF-α and ox-LDL in serum and brain are shown in Fig. 4.

Compared to the CD group, the levels of IL-1, TNF-α, and ox-LDL in the serum and brain in the CCT group were significantly increased (*P* < 0.05). Between-group comparison showed that the levels of serum and brain IL-1, TNF-α, and ox-LDL in CCT + LYCO (25, 45, 65, and 85 mg·kg^− 1^) groups were lower than those in CCT groups (*P* < 0.05). CCT and CCT + LYCO (5 mg·kg^− 1^) groups did not show any significant alterations in serum and brain IL-1, TNF-α, or ox-LDL (*P* > 0.05). There also were no significant differences in serum or brain IL-1 levels between the CCT and CCT + F groups (*P* > 0.05).

### Brain LDLR and NGF measurement

**Fig. 5:**
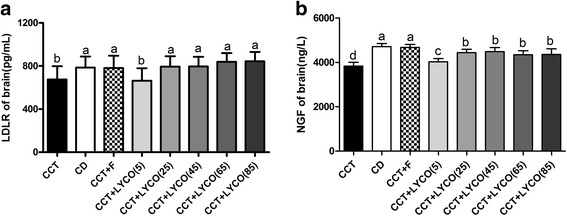
Brain LDLR and NGF in different groups at the end of the experiment. *n* =10, values are expressed as the mean ± SD, and bars without a common superscript letter differ significantly (*P* < 0.05). Kruskal-Wallis *H* test or ANOVA results showed: **a** LDLR in brain: *H* = 54.178, *P* = 0.000; **b** NGF in brain: *F* = 30.094,*P* = 0.000

The levels of LDLR and NGF in brain are shown in Fig. 5.

The levels of cerebral LDLR and NGF were lower in the CCT group than the control group (*P* < 0.05), but there was no significant difference in brain LDLR level among rats in CCT and CCT + LYCO (5 mg·kg^− 1^) groups. The levels of brain LDLR and NGF in CCT + LYCO (25, 45, 65, and 85 mg·kg^− 1^) groups were higher than in the CCT group (*P* < 0.05), but there was no significant difference between them (*P* > 0.05).

### Neurotransmitters and their receptors

**Fig. 6:**
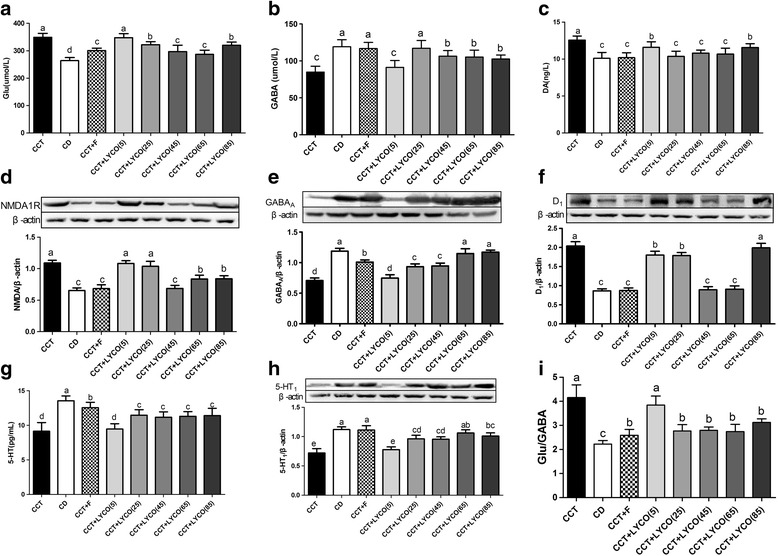
Neurotransmitters and their reports in different groups at the end of the experiment. *n*=10, values are expressed as the mean ± SD, and bars without a common superscript letter differ significantly (*P* < 0.05). Kruskal-Wallis *H* test or ANOVA results showed: **a** Glu: *F*_Glu_ = 41.888, *P* = 0.000; **b** GABA: *F*_GABA_ = 21.116, *P* = 0.000; **c** DA: *F*_DA_ = 16.770, *P* = 0.000; **d** NMDA1R: *H*_NMDA1R_ = 69.397, *P* = 0.000; **e** GABA_A_: *H*_GABAA_ = 70.451, *P* = 0.000; **f** D_1_: *H*_D1_ = 65.429, *P* = 0.000; **g** 5-HT: *F*_5-HT_27.851, *P* = 0.000; **h** 5-HT_1_: *H*_5-HT1_ = 65.416, *P* = 0.000; **i** Glu/GABA: *H* = 65.028, *P* = 0.000

At the end of the experiment, The expression of neurotransmitters (Glu, GABA, 5-HT, DA) and their reptors (NMDA1R, GABA_A_, 5-HT_1_, D_1_) are shown in Fig. 6.

The levels of Glu and DA as well as the expression of NMDA1R and D_1_ were higher in the CCT group than the control group (*P* < 0.05), while the levels of GABA and 5-HT and expression of GABA_A_ and 5-HT_1_ were lower (*P* < 0.05); the Glu/GABA ratio was also larger (*P* < 0.05). CCT and CCT + LYCO (5 mg·kg^− 1^) groups exhibited no significant alteration in the levels of Glu, GABA, and DA or the expression of NMDA1R, D_1_, and 5-HT_1_ (*P* > 0.05). The levels of Glu and DA and the ratio of Glu/GABA were lower compared to the CCT group, while the levels of GABA and 5-HT and the expression of GABA_A_ and 5-HT_1_ were higher in CCT + LYCO (25, 45, 65, and 85 mg·kg^− 1^) groups (*P* < 0.05). There were no significant differences in DA levels in CCT + LYCO (25, 45, 65, and 85 mg·kg^− 1^) groups or GABA in LYCO (25 mg·kg^− 1^) groups, nor in the expression of D_1_ in CCT + LYCO (45 and 65 mg·kg^− 1^) groups or GABA_A_/5-HT_1_ in CCT + LYCO (65 mg·kg^− 1^) groups compared to the control group (*P* > 0.05).

### Proteins related to apoptosis

**Fig. 7:**
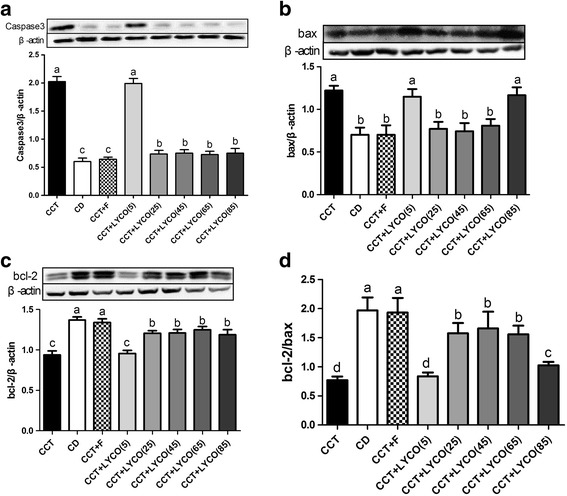
Pro-apoptotic Caspase-3, bax and anti-apoptotic bcl-2 in different groups at the end of the experiment. *n*=10, values are expressed as the mean ± SD, and bars without a common superscript letter differ significantly (*P* < 0.05). Kruskal-Wallis *H* test results showed: **a** Caspase-3: *H* = 60.967, *P* = 0.000; **b** bax: *H* = 60.579, *P* = 0.000; **c** bcl-2: *H* = 68.025, *P* = 0.000; **d** bcl-2/b:ax *H* = 66.029, *P* = 0.000

At the end of the experiment, The expression of pro-apoptotic Caspase-3, bax and anti-apoptotic bcl-2 are shown in Fig. 7.

The expression of Caspase3 and bax in the brain of CCT group rats were higher than in the control group (*P* < 0.05). The expression of bcl-2 and the bcl-2/bax ratio were also lower than the control group (*P* < 0.05). CCT, CCT + F, CCT + LYCO (5 mg·kg^− 1^), and CD groups showed no significant alterations in the expression of Caspase3, bax, or bcl-2 nor in the ratio of bcl-2/bax (*P* > 0.05). The expression of Caspase3 in CCT + LYCO (25, 45, 65, and 85 mg·kg^− 1^) groups were lower than in the CCT group (*P* < 0.05). By contrast, bcl-2 expression and bcl-2/bax ratio were higher than in the CCT group (*P* < 0.05). The lowest bcl-2/bax ratio was measured in the CCT + LYCO (55 mg·kg^− 1^) group (*P* < 0.05).

### Morphologic changes in hippocampus

**Fig. 8:**
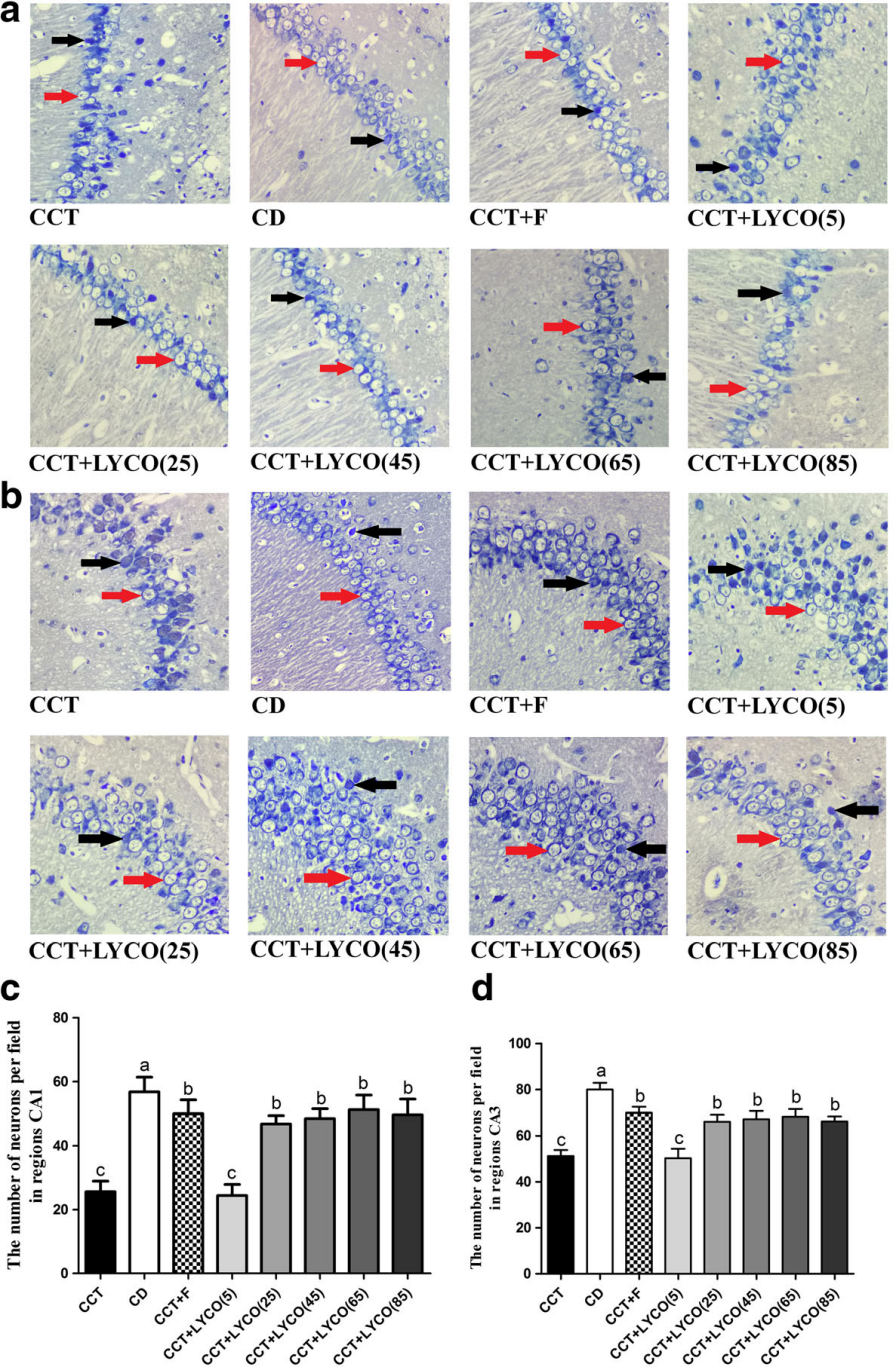
Morphologic changes of hippocampus stained by Nissl staining using a light microscope (magnification 400×) and counting the results. The red arrow shows the normal nurons, and the black arrow shows the degenerated neurons.*n*=10, values are expressed as the mean ± SD. Bars without a common superscript letter differ significantly (*P*<0.05). **a** Photomicrographs of the CA1 obtained via Nissl staining. **b** Photomicrographs of the CA3 obtained via Nissl staining. The number of neurons per field in regions CA1 (**c**) and CA3 (**d**) of hippocampus. *H*_CA1_ = 57.007, *P* = 0.000; *H*_CA3_ = 62.606,*P* = 0.000

Nissl staining was used to detect the morphologic changes of hippocampus in rats and the count of neurons in regions CA1 and CA3 were measured (there sections from each group were selected and counted using Image-ProPlus software) [28], and the results are shown in Fig. 8.

Nissl staining results observed under a high-power lens indicated that CCT group rats had a decreased number of hippocampal pyramid cells compared to the CD group; the neurons were also disorderedly arranged and were mostly stained deeply forming triangular shapes with degeneration and karyopyknosis. In CD group rats, conversely, there were large amounts of hippocampal pyramid cells arranged tightly and regularly with clear profiles and distinct demarcation from the surrounding tissue. The morphological changes in CCT + F group pyramid cells were similar to those in the control group plus a small amount of degenerative neurons. In the CCT + LYCO (5 mg·kg^− 1^) group, the hippocampal neurons were arranged loosely with substantial degeneration and necrosis, similar to the CCT group. The morphological changes in rat hippocampi after treatment with CCT + LYCO (25, 45, 65, and 85 mg·kg^− 1^) were similar: Neurons were tightly arranged and in greater abundance than those in the CCT group samples, and degenerative neurons and Nissl bodies were clearly observed.

The neurons in hippocampal CA1 and CA3 areas decreased in the CCT group compared to the CD group (*P* < 0.05). There was no significant difference in the number of hippocampal neurons between CCT group and CCT + LYCO (5 mg·kg^− 1^) groups (*P* > 0.05). The number of neurons in hippocampal CA1 and CA3 areas in CCT + LYCO (25, 45, 65, and 85 mg·kg^− 1^) group and positive drug groups were higher than those in the model group (*P* < 0.05), which was lower than that in the control group (*P* < 0.05).

### Correlation coefficient matrix of serum lipids and brain parameters

**Fig. 9:**
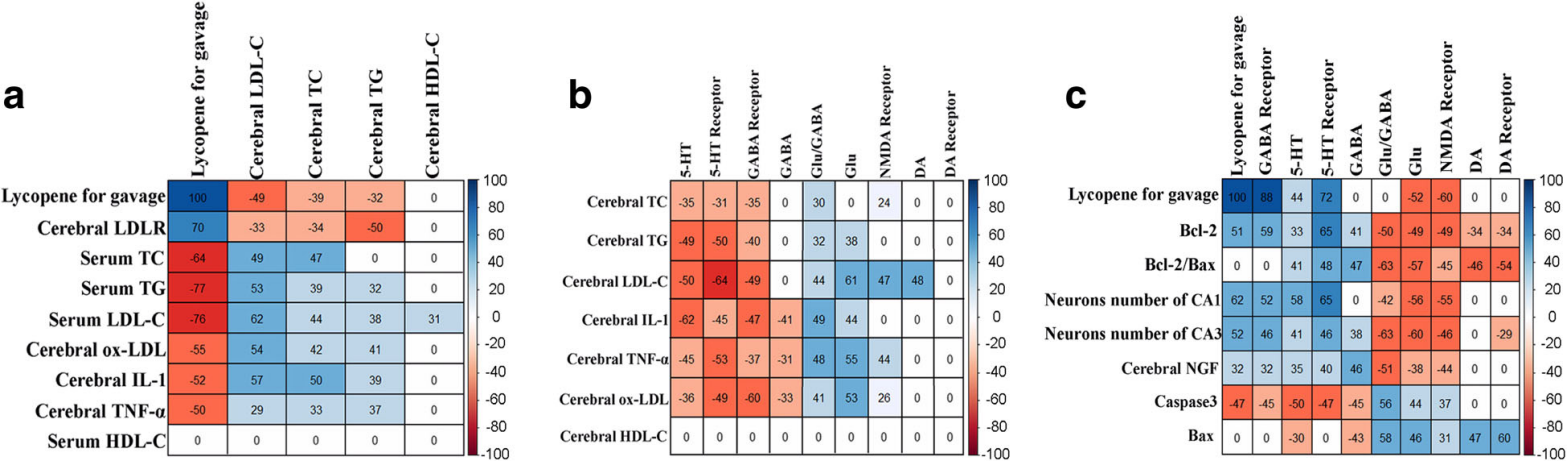
Correlation coefficient matrix of serum lipid and brain parameters. **a** Correlation coefficient matrix between plasma lipids and cerebral parameters. **b** Correlation coefficient matrix of cerebral parameters and neurotransmitters. **c** Correlation coefficient matrix between cerebral apoptosis related proteins and neurotransmitters. Colors indicate directionality (blue denotes positive; red denotes negative) and the strength of the association

We conducted a correlation analysis of serum lipid and brain parameters of lycopene CCT + LYCO (5, 25, 45, 65 and 85 mg·kg^− 1^) groups, and represent the outcome in a matrix diagram [29], showed in Fig. 9.

The dose of lycopene for gavage was positively correlated with TC, TG, and LDL-C in the serum and brain; it was also positively correlated with ox-LDL, IL-1, TNF-α, Glu, NMDA1R, and Caspase-3 in the brain and negatively correlated with cerebral LDLR, NGF, 5-HT_1_, GABA_A_, and neuron quantity in hippocampal CA1 and CA3 areas (*P* < 0.05). In addition, brain TC, TG, and LDL-C were all positively correlated while brain ox-LDL, IL-1, and TNF-α were negatively correlated with LDLR in the brain (*P* < 0.05). The levels of ox-LDL, IL-1, and TNF-α in the brain was positively correlated with the Glu/GABA ratio, but negatively correlated with 5-HT, 5-HT_1_, and GABA_A_ (*P* < 0.05). The expression of Caspase-3 in the brain was positively correlated with Glu and NMDA1R, but negatively correlated with GABA, 5-HT, GABA_A_, and 5-HT_1_ (*P* < 0.05). The bcl-2/bax ratio and quantity of neurons in hippocampal area CA1 were also negatively correlated with Glu/GABA, NMDA1R, and DA_1_ but positively correlated with GABA, 5-HT, and 5-HT_1_ (*P* < 0.05).

## Discussion

Lipid metabolism disorders, inflammatory reactions, and oxidative stress caused by hyperlipidemia can damage the brain tissue [30, 31]. Lycopene is a natural carotenoid which has strong antioxidant and anti-inflammatory effects [32]. Studies have shown that long-term use of lycopene can reduce the mortality of patients with AD, and that the material exerts protective effects on the brain [19]. The structure and function of the central nervous system are highly complex. The neurotransmitter plays a crucial role in the process of neurochemical signaling, and thus may play an important role in the protective effects of lycopene on brain injury. The results of our previous experiments showed that hyperlipidemia had damage on the brain of both female and male rats [22, 23]. Therefore, two gender rats were selected as the subjects of this study.

We established the hyperlipidemia model with reference to the Deepa modeling method (normal rat chow supplemented with 4% cholesterol, 1% cholic acid, and 0.5% thiouracil, CCT diet) [25]. After 1 week of consuming the CCT, the levels of serum TC, TG, and LDL-C increased 3.40, 2.29, and 3.00 fold in our rats (*P* < 0.05), respectively, while the levels of serum HDL-C decreased 0.78 fold (*P* < 0.05). Repeated measures analysis of variance showed that the severity of hyperlipidemia in the CCT group increased with the prolonging of feeding time (*P* < 0.05), which consistents with our previous study [23]. In regards to the physiological state, brain cholesterol is mainly synthesized in situ by astrocytes via the Kandutsch-Russel pathway [33], and can be transformed into 24S–oxycholesterol under 24S–hydroxylase into the liver for catabolism [34]. Excessive cholesterol can be ingested and stored in the lipid raft by LDLR on the neruons [35]. These processes function together to maintain the dynamic balance of brain cholesterol.

In this study, we found that the levels of cerebral TC, TG, and LDL-C increased while the LDLR decreased in the rat brain after 4 weeks on consuming the CCT (*P* < 0.05). Cerebral TC, TG, and LDL-C was positively correlated with serum TC, TG, and LDL-C (*P* < 0.05) according to the correlation analysis; this suggests that hyperlipidemia can reduce the expression of LDLR in the brain, inhibit the transportation of brain cholesterol, and destroy the balance of brain lipid metabolism.

Studies have shown that hyperlipidemia can stimulate microglia, astrocytes, and other central immune cells to secrete a variety of cytokines (IL-1, TNF-α) exerting direct and toxic effects on the brain [36, 37]. These effects lead to brain energy metabolism disorders which yield a variety of reactive oxygen free radicals, promote LDL oxidation modification to form ox-LDL, and ultimately destroy the structures of cells via apoptosis [38, 39]. The biochemical analysis focused on the whole brain in order to research the effects of several factors on the brain. Meanwhile, the morphological analysis was used to determine the change of neurons in the anti-injury of lycopene induced by hyperlipidemia, which verifying the results of biochemistry. Hippocampus is an important region of brain neurons, and it’s very sensitive to cerebral ischemia, damage and other stimuli. Also, there are lots of nerve circuits in CA1 and CA3 regions of hippocampal. It could fully represent the damage of cerebral nerve. In this study, the levels of serum IL-1, TNF-α, and ox-LDL in CCT group rats increased (*P* < 0.05) while the bcl-2/bax ratio and quantity of neurons in CA1 and CA3 areas decreased compared to the CD group (*P* < 0.05). Correlation analysis data (Fig. 9) indicated that TC, TG, and LDL-C levels were positively correlated with IL-1, TNF-α, and ox-LDL in the brain (*P* < 0.05), while the levels of brain IL-1, TNF-α, and ox-LDL were negatively correlated with bcl-2/bax ratio and neuron quantity in hippocampal CA1 and CA3 areas (*P* < 0.05). These results suggest that hyperlipidemia can affect the lipid metabolism of the brain, and cause damage to the brain by enhancing inflammatory and oxidative reactions which induce neuronal apoptosis.

Lycopene, as discussed above, is a natural carotenoid with a strong anti-inflammatory and oxidative capacity that has been shown to inhibit the activity of cholesterol synthase and to regulate lipid balance [40–42]. Epidemiological studies have shown that long-term intake of lycopene-rich foods reduces the risk of cognitive dysfunction [43]. Experimental studies have also shown that lycopene can inhibit mitochondrial oxidative damage, reduce the secretion of Aβ to protect neurons, and prevent the occurrence of Huntington’s disease (HD), AD, and other neurodegenerative diseases [44, 45]. The present study suggests that TC, TG, LDL-C, IL-1, TNF-α, and ox-LDL levels in the serum and brain of rats in the CCT + LYCO (25, 45, 65, and 85 mg·kg^− 1^) group were lower than those in the CCT group, while LDLR level was higher (*P* < 0.05). The dose of lycopene for gavage was also not only negatively correlated with TC, TG, and LDL-C levels in the serum and brain, but also negatively correlated with ox-LDL, IL-1, and TNF-α levels while being positively correlated with LDLR in the brain (*P* < 0.05). TC, TG, and LDL-C levels in the brain were positively correlated with ox-LDL, IL-1, and TNF-α levels but negatively correlated with brain LDLR (*P* < 0.05). This suggests that lycopene can prevent increases in serum TC, TG, and LDL-C, enhance the activity of brain LDLR, promote cholesterol transport, and indirectly adjust the brain TC, TG, and LDL-C levels; further, it can reduce the production of ox-LDL, IL-1, and TNF-α and alleviate the injury due to inflammatory and oxidative stress in hyperlipidemia.

The central nervous system consists of neurons and glial cells which mainly rely on neurotransmitters for information transfer. There are hundreds of known neurotransmitters including amino acids, monoamines, cholines, peptides, and purines. Hyperlipidemia can disrupt the balance of brain neurotransmitters [11, 12], and is closely related to a variety of neurodegenerative diseases [46, 47], in which the balance of amino acids and monoamine neurotransmitters plays an significant role in injuring the brain. Brain amino acid neurotransmitters mainly include excitatory amino acids (EAA) such as Glu and inhibitory amino acids (IAA) such as GABA. Glu has a strong excitatory effect on neurons; over-excited neurons can produce “excitotoxic effects” [48]. When Glu binds to its NMDA1 receptor, it can cause an influx of Ca^2+^ from extracellular areas and activate a variety of cytotoxic enzymes to induce lipid peroxidation and destroy important structures such as neuronal lipid membranes, proteins, and DNA [49]. Damage to cognitive function in mice with CCT was associated with abnormal Glu metabolism in the hippocampus in a previous study [12]. In contrast to Glu, GABA binds to GABA_A_ receptors to open Cl^−^ channels and hyperpolarize the postsynaptic membrane, resulting in inhibitory post synaptic potential (IPSP) that decreases excitability and protects the neurons [50]. GABA can also inhibit the inflammatory response in mice fed with high-fat diets [51]. These two kinds of amino acids often coexist due to their different mechanisms; the Glu/GABA ratio is often used to reflect the objective function of both simultaneously.

In the study, the Glu, Glu/GABA, and Caspase-3 in rats in CCT + LYCO (25, 45, 65 and 85 mg·kg^− 1^) groups were lower than those in the CCT group (*P* < 0.05), while GABA and GABA_A_ expression as well as quantity of neurons in hippocampal CA1 and CA3 areas were higher than those in the CCT group (*P* < 0.05). Correlation analysis showed that the lycopene dose for gavage was negatively correlated with Glu, NMDA1 receptor, and Caspase-3 levels in the brain (*P* < 0.05) but positively correlated with GABA_A_ and bcl-2 expressions as well as CA1 and CA3 area neuron quantities (*P* < 0.05). Brain Glu, Glu/GABA, and NMDA1R were positively correlated with ox-LDL, TNF-α, Caspase-3, and bax in the brain but negatively correlated with bcl-2 expression, bcl-2/bax ratio, and the quantity of neurons in hippocampal CA1 and CA3 areas (*P* < 0.05). GABA level and GABA_A_ expression were also negatively correlated with IL-1, TNF-α, and ox-LDL in the brain (*P* < 0.05) but positively correlated with the expression of bcl-2 and neuron quantity in the CA3 area (*P* < 0.05). These results altogether suggest that lycopene can not only inhibit the secretion of Glu and the expression of NMDA1 receptors, thus weakening the excitotoxicity of amino acid neurotransmitters, downregulating the expression of Caspase-3, but also enhance the inhibitory effect of GABA_A_ receptors to maintain a balanced bcl-2/bax ratio.

Monoamine neurotransmitters including norepinephrine (NE), epinephrine (E), DA, 5-HT, and others are involved in the regulation of sensory, sleep, learning/memory, and cardiovascular activities. When the D1 receptor is excited, the stimulating adenylate cyclase G proteinit (Gs) can be activated, which exerts physiological effects by phosphorylation of the protein [52]. DA can be transformed into reactive oxide through a metabolic process which activates the c-Jun N-terminal kinase (JNK) signaling pathway to induce neuronal apoptosis [53]. Studies have shown that high-fat diet can inhibit the degradationrat of DA so as to elevate the level of DA in brain [54]. After activation of the 5-HT_1_ receptor, it can be coupled to the Gi protein to inhibit the production of cAMP and promote the outflow of K^+^, ultimately producing a prominent post inhibitory effect. 5-HT level decreased in the brains of mice fed with high fat diets [55], suggesting that it may be related to neurodegenerative diseases. It has been shown that the dysregulation of nerve growth factor (NGF) signal transduction may be responsible for the pathogenesis of neurodegenerative diseases such as AD and HD [56, 57], which plays an important role in the development, differentiation, apoptosis, and neurotransmitter transmission of the cells.

Compare with the CCT group, when administered various doses of lycopene, the levels of NGF, 5-HT, and the expression of 5-HT_1_ receptors and quantity of neurons in the same hippocampal areas were higher in CCT + LYCO (25, 45, 65, and 85 mg·kg^− 1^) groups (*P* < 0.05); their level of DA was also relatively low (*P* < 0.05). Correlation analysis showed that the dose of lycopene for gavage was positively associated with the levels of NGF, 5-HT, 5-HT_1_ receptor expression, and neuron quantity in hippocampal CA1 and CA3 areas (*P* < 0.05), while there was no significant association with the DA or D_1_ receptor (*P* > 0.05). In addition, 5-HT and 5-HT_1_ receptors were negatively correlated with the brain IL-1, TNF-α, ox-LDL, and Caspase-3 expression, but positively correlated with bcl-2/bax ratio and quantity of neurons in hippocampal CA1 and CA3 regions (*P* < 0.05). Our results confirmed that lycopene protects against damage by promoting the release of NGF and 5-HT, enhancing the inhibitory effect of the 5-HT_1_ receptor, downregulating the expression of Caspase-3, and upregulating the expression of bcl-2 to maintain hippocampal neurons with normal morphology.

## Conclusions

In conclusion, hyperlipidemia is a risk factor for neurodegenerative diseases such as AD and Parkinson’s disease. Central nervous system diseases are closely related to neurotransmitter metabolism disorder. Lycopene exerts a protective effect on the brain induced by hyperlipidemia. It can inhibit any elevation in serum TC, TG, and LDL-C in hyperlipidemic rats, and affect the levels of TC, TG, and LDL-C in the brain indirectly to promote the secretion NGF, leading to a reduction in ox-LDL, IL-1, and TNF-α in the brain. Reduction in oxidation and inflammation inhibits Glu release, which weakens excitatory nerve toxicity while downregulating pro-apoptotic Caspase-3. Lycopene also exerts anti-injury effects by promoting the release of inhibitory neurotransmitters GABA and 5-HT and by upregulating the anti-apoptotic bcl-2. Taken together, our results suggest that lycopene protects against brain damage induced by hyperlipidemia by regulating Glu, GABA, 5-HT, and other neurotransmitters. To this effect, lycopene may have the protective effect against neurodegenerative diseases induced by hyperlipidemia.

## Abbreviations

5-HT: 5-hydroxytryptamine
AD: Alzheimer’s disease
Caspase-3: Cysteine aspartate specific protesae-3;
CD: Chow diet
CMC-Na: Carboxymethylcellulose sodium
COD-PAP: Cholesterol oxidase-peroxidase-4-aminoantipyrine
DA: Dopamine
EAA: Excitatory amino acids
GABA: Gamma aminobutyric acid
Glu: Glutamic acid
GPO-PAP: Glycerol phosphate oxidase-peroxidase-4-aminoantipyrine;
HD: Huntington’s disease
IAA: Inhibitory amino acids
LDLR: Low-density lipoprotein receptor
LYCO: Lycopene
NGF: Nerve growth factor
NMDA1R: N-methyl-D-aspartate
ox-LDL: Oxidized low density lipoprotein
TC: Total cholesterol
TG: Total triglycerides
