# Correction to: Mechanisms of multiple neurotransmitters in the effects of Lycopene on brain injury induced by Hyperlipidemia

**DOI:** 10.1186/s12944-018-0700-1

**Published:** 2018-04-03

**Authors:** Weichun Yang, Ziyi Shen, Sixian Wen, Wei Wang, Minyu Hu

**Affiliations:** Department of Nutrition and Food Hygiene, Xiangya School of Public Health, Central South University, 110 Xiangya Road, Changsha, 410078 China

## Correction

Following publication of the original article [1] it came to the attention of the Research Integrity Group that the following corrections were required:

– In the “Consent for publication” section the sentence “All co-authors and participants have given their consent for publication of this article in Lipids in Health and Disease” should be replaced with “Not applicable”

– The section titled “Competing of interests” should be corrected to “Competing interests”

– The title of the section labeled “Author’ contributions” should be changed to “Authors’ contributions”

– The sentence “None of the authors reported a conflict of interest related to this study” should be removed from the end of the “Authors’ contributions” section.

The original article has been corrected.
